# Obstructive Sleep Apnea Alters Sleep Stage Transition Dynamics

**DOI:** 10.1371/journal.pone.0011356

**Published:** 2010-06-28

**Authors:** Matt T. Bianchi, Sydney S. Cash, Joseph Mietus, Chung-Kang Peng, Robert Thomas

**Affiliations:** 1 Neurology Department, Massachusetts General Hospital, Boston, Massachusetts, United States of America; 2 Division of Interdisciplinary Medicine and Biotechnology, Beth Israel Deaconess Medical Center, Boston, Massachusetts, United States of America; 3 Sleep Division, Beth Israel Deaconess Medical Center, Boston, Massachusetts, United States of America; Cuban Neuroscience Center, Cuba

## Abstract

**Introduction:**

Enhanced characterization of sleep architecture, compared with routine polysomnographic metrics such as stage percentages and sleep efficiency, may improve the predictive phenotyping of fragmented sleep. One approach involves using stage transition analysis to characterize sleep continuity.

**Methods and Principal Findings:**

We analyzed hypnograms from Sleep Heart Health Study (SHHS) participants using the following stage designations: wake after sleep onset (WASO), non-rapid eye movement (NREM) sleep, and REM sleep. We show that individual patient hypnograms contain insufficient number of bouts to adequately describe the transition kinetics, necessitating pooling of data. We compared a control group of individuals free of medications, obstructive sleep apnea (OSA), medical co-morbidities, or sleepiness (n = 374) with mild (n = 496) or severe OSA (n = 338). WASO, REM sleep, and NREM sleep bout durations exhibited multi-exponential temporal dynamics. The presence of OSA accelerated the “decay” rate of NREM and REM sleep bouts, resulting in instability manifesting as shorter bouts and increased number of stage transitions. For WASO bouts, previously attributed to a power law process, a multi-exponential decay described the data well. Simulations demonstrated that a multi-exponential process can mimic a power law distribution.

**Conclusion and Significance:**

OSA alters sleep architecture dynamics by decreasing the temporal stability of NREM and REM sleep bouts. Multi-exponential fitting is superior to routine mono-exponential fitting, and may thus provide improved predictive metrics of sleep continuity. However, because a single night of sleep contains insufficient transitions to characterize these dynamics, extended monitoring of sleep, probably at home, would be necessary for individualized clinical application.

## Introduction

Numerous endogenous and exogenous factors influence whether sleep or wake is achieved, how long a given state is maintained, and the reasons sleep architecture may become fragmented [1], [2], [3], [4]. Much effort has been invested in attempts to correlate various polysomnogram (PSG) metrics with daytime symptoms, with the goal of understanding (and promoting) those aspects of sleep that contribute most to its recuperative properties. However, correlations between daytime sleepiness and PSG metrics are not always straightforward, due in part to inter-subject variability, the subjective nature of the clinical complaints, and variations in an individual's tolerance to sleep disruption. The commonly employed Epworth Sleepiness Scale (ESS), for example, correlates with subjective complaints of sleepiness but not with objective measures obtained from Multiple Sleep Latency Tests [5], [6].

Although the ESS score was correlated with the severity of obstructive sleep apnea (OSA) in the large Sleep Heart Health Study (SHHS) database, the absolute changes were small and even the most severe OSA group had scores within the normal range (<10)[7]. Other measurements have also been investigated as predictors of daytime sleepiness, including fragmentation [8], autonomic arousals [9], and EEG measurements of cortical arousals associated with respiratory events in OSA patients [10]. A meta-analysis of the relationship between sleep fragmentation and daytime function suggested the importance of the percentage of stage NREM1 which may inversely relate to sleep continuity [11]. However, other reports show little relationship of stage NREM1 proportions with fragmentation [12], [13]. Percentage or proportion of a state does not contain information about the number of transitions, or fragmentation, which may contribute to some of the differences in reporting.

Survival analysis applied to sleep bout durations, in which lifetime refers to the length of time spent in a given stage, indicates decreased stability of sleep in OSA patients in proportion to severity of disease[14]. Importantly, when sleep bout duration is considered in this dynamic sense (the distribution of time spent in a state), OSA was shown to alter sleep architecture despite unchanged “summary” PSG metrics, such as stage percentages or total sleep time[15]. Decreased sleep stability, as measured by transition probabilities and survival analysis of sleep runs (stage-independent), was also demonstrated in Chronic Fatigue Syndrome patients[16], [17]. Transition probability analysis also demonstrated that sleep fragmentation seen in elderly patients was due to more frequent awakenings, but the wake bouts were unchanged in duration, suggesting that the transition back to sleep was unaffected by age[18]. These studies increasingly suggest that standard metrics of sleep architecture and fragmentation/arousal can be complemented by assessing the distribution of stage durations and transition probabilities, in hopes of providing better correlations with disease states and clinical symptoms.

Given the increasing realization that the percentage of time in a given stage may be less important than their distribution dynamics across the night, several groups have considered sleep architecture in terms of the statistical distributions of sleep stage durations. In fact, measurements of sleep in rodents, cats, and humans suggested that the duration of sleep bouts appear to follow mono-exponential kinetics, with species-specific time constants governing the time spent in a given bout of sleep[19], [20]. However, the mono-exponential models often did not fit well over the entire range of bout durations, suggesting that a more complex model may be required. Wake bout duration kinetics remain controversial, being described as either an exponential process[21], or a power law process[19], [20], [22]. Whereas exponential distributions imply stochastic (probabilistic) state transitions characterized by a time constant of “decay” or exit from a particular state, a power law distribution implies a so-called scale-free (or fractal) process, with no characteristic time scale of measurement. Connecting the rapidly growing basic science understanding of sleep- and wake- promoting brain centers to the behavioral manifestation of arousal state transitions requires improved understanding of transition dynamics. To accomplish this, we analyzed hypnograms from the large SHHS database, to answer the following questions: 1) what is the best model of sleep stage transition dynamics; 2) is a single night of data sufficient for fitting and model discrimination; 3) how does sleep fragmentation, such as that caused by OSA, alter the temporal dynamics?

Our results demonstrate that multi-exponential fitting is superior to routine mono-exponential fitting, but also show that a single night of sleep contains insufficient transitions to characterize these dynamics. Wake bout distributions may be fitted by either a multi-exponential or a power law model. OSA alters the dynamics by accelerating the decay of REM and NREM bout durations, reflecting sleep fragmentation despite unchanged summary metrics of state percentages.

## Results

### Characterizing sleep architecture dynamics

The summary statistics obtained from the PSG data for the three patient groups are presented in Table 1. Note that despite large differences in the AHI (1.8 vs 8.6 vs 47.4) and arousals, the percent sleep efficiency and the N1 percentages were not different. The differences in percentages of REM, N2 and N3 sleep did reach significance: N2 showed a small increase in percentage across mild and severe apnea; REM and N3 showed the inverse pattern.

**Table 1:** Group demographics and sleep-related data.

Variable	Control	Mild OSA	Severe OSA	ANOVAF_(2, 1207)_, p	Tukey
N	376	496	338		
Age	68.2±6.3	63.8±10.3	63.7±10.5	30.43, <0.001	1>2, 3
Sex (% males)	35.6	60	70.7	* 95.5, <0.001	
Race (% Caucasian)	85.4	73.8	74.3	*7.91, <0.001	
BMI (kg/m^2^)	26.3±4	30.1±5.4	32.4±5.8	128.5, <0.001	3>2,12>1
Anti-HTN use (%)	0	53.6	54.4	*178.8, <0.001	
Systolic BP (mm Hg)	125.2±17.2	129.1±19.2	131.7±19.4	10.7, >0.001	2>13>13>2
Diastolic BP (mm Hg)	70.6±10.2	74.2±10.6	78.2±13.1	40.4, <0.001	3>1,23>2
Diabetes (%)	0	9.5	5.9	* 3.54, 0.06	
Angina (%)	0	9.5	9.5	* 51, <0.001	
Myocardial infarction (%)	0	10.9	7.1	* 47.7, <0.001	
CHF (%)	0	2.6	1.8	* 24.9, <0.001	
ESS	4.9±2.2	13.1±2.8	9.8±4.9	610.6, <0.001	2>1,33>2
Sleep efficiency (%)	81.2±11.1	81.7±10.2	79.4±11.2	2.38, 0.09	N.A.
Sleep Latency (min)	22.7±23.9	23.1±22.5	22.9±21.4	0.02, 0.9	N.A.
Total sleep time (min)	360±64.8	326.5±61.9	344.2±61.7	53,9, <0.001	1>2,33>2
N1 (%)	5.8±4.2	5.8±4.3	6.4±5.1	2.5, 0.08	N.A.
N2 (%)	54.9±11.9	59.3±10.7	63.1±11.9	44.7, <0.001	3>1,22>1
N3 (%)	19±12.3	15.5±11.4	13.2±11.2	22.2, <0.001	1>2,32>3
REM (min)	20.3±5.8	19.4±6	17.3±6.4	23.2, <0.001	1>32>3
REM latency (min)	85.2±53.7	86±56.4	102.6±69.3	8.81, <0.001	3>1,2
Arousal index – total	17.3±8.6	19±8.9	36.4±16.4	302.3, <0.001	3>1,2
Arousal index – NREM only	18.2±9.3	19.7±9.6	38±17.3	284.2, <0.001	3>1,2
Arousal index – REM only	13.6±8.7	15.7±9.7	27.3±16.8	135.1, <0.001	3>1,22>1
RDI	23.6±14	36.3±13.5	71.9±17.2	1020.2, <0.001	3>1,22>1
AHI	1.8±1.4	8.6±2.5	47.4±16.4	2795.3, <0.001	3>1,22>1

*Pearson Chi^2^ 95.5.

Values are mean ± SD. BMI, body mass index; CHF, congestive heart failure; BP, blood pressure; HTN, hypertension; ESS, Epworth Sleepiness Scale.

**Figure 1:**
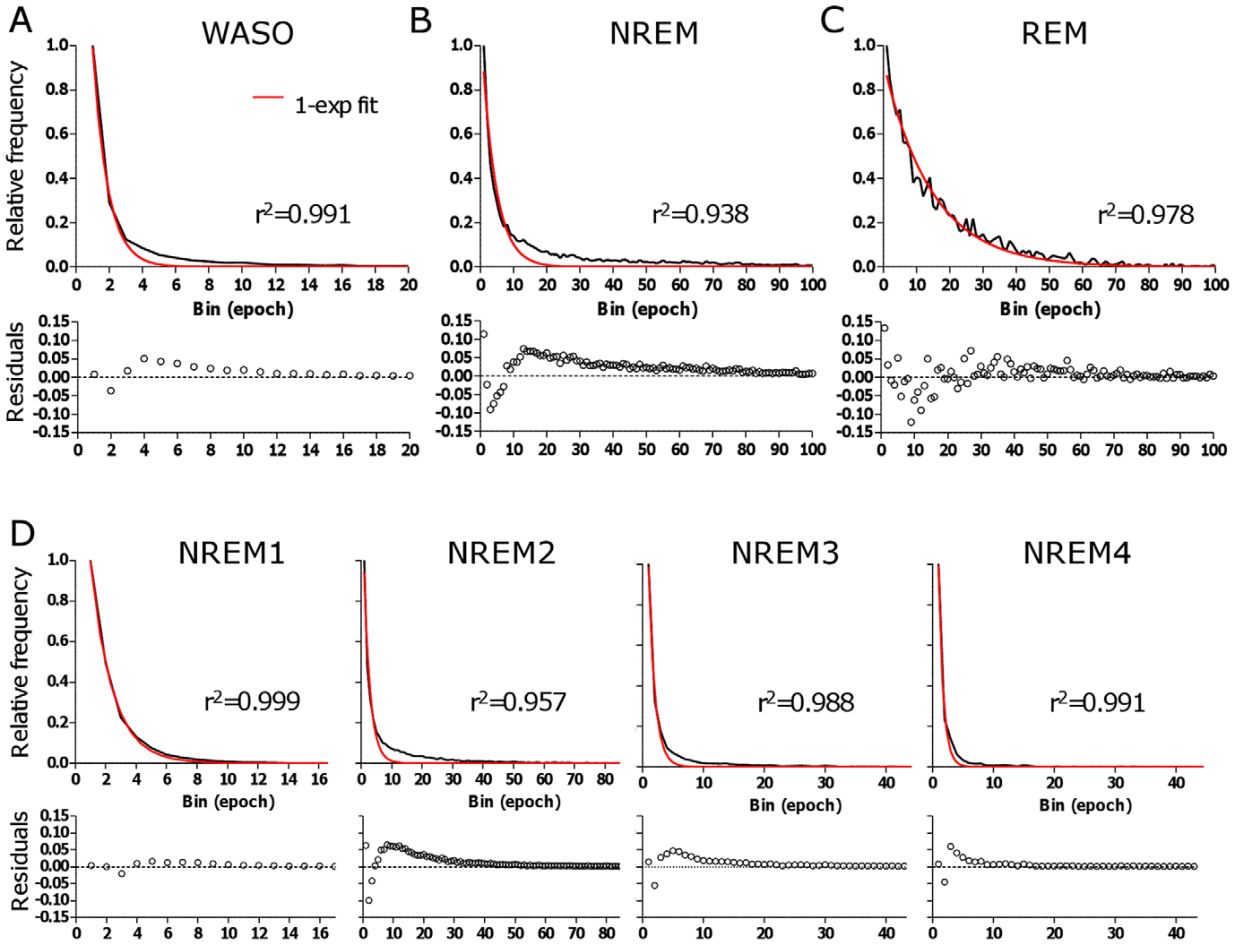
Frequency histogram analysis of bout durations in the control group. The relative frequency of bouts in the control group is plotted against the duration of bouts (in bins of 30-second increments on the x-axis) for WASO (A), NREM (B), REM sleep (C) and sub-stages of NREM sleep (D) bouts. In each panel, the best fit single-exponential function (red) is overlaid, and the residuals (difference between data and fit) are plotted beneath each histogram.

We examined the sleep architecture dynamics by focusing on the distribution of bout durations of WASO, REM, and NREM sleep. This was accomplished by generating frequency histograms for each stage, a common technique that allows visualization of data distributions, as well as fitting with functions such as exponential and power law models. These plots are obtained by collecting the relative number of events (y-axis) occurring in each “bin” (x-axis), defined here in single epoch increments of 30 seconds each. Figure 1 illustrates the frequency histograms of WASO, NREM sleep, and REM sleep bout durations for the control group. In these panels, “NREM” does not distinguish sub-stage composition (that is, transitions within NREM sub-stages are ignored, and NREM is taken as a single stage). Exponential fitting of the duration distributions for each stage was performed (see Methods). In each plot, the best fit single exponential function is overlaid for visual inspection of goodness of fit. In addition, the r^2^ values and the residuals (subtracting the fitted line from the data) are shown in each case. Despite the high r^2^ values (0.93–0.99), visual inspection of the WASO, overall NREM sleep and NREM sub-stage plots shows that the best single exponential function is not adequate: it emphasizes the rapid decay phase (consisting of brief events, which were more commonly observed) but entirely misses the longer duration events (that occur less frequently). Therefore, despite its common use (see, for example, ref [22]), r^2^ values as a measure of goodness of fit should be used with caution for such distributions. We show the traditional four NREM sub-stages to determine possible differences in dynamics that could explain the multi-exponential pattern seen in the global NREM metric, although clinically it has been determined that stages 3 and 4 should be considered a single state (called N3). Therefore, for the analysis shown in Figure 1, we assessed additional transitions not currently recognized in the clinical scoring guidelines. The number of NREM4 bouts was ∼2% of total bouts in this control cohort.

The residuals (Figure 1) provide an alternative view of how different the fitted line is from the actual data, and it also reveals why the r^2^ values can appear statistically favorable (close to 1.0) despite being visually sub-optimal. Although it appears that the mono-exponential curve matches the fast decay but ignores the slow decay, in fact the residuals demonstrate the opposite: systematic deviations between the fitted line and actual data were most prominent during the fast decay (short duration bins). The absolute y-axis magnitude of the long duration “tail” is small, and thus the residual between the fit and the data is small for the majority of bin sizes, even though the fit completely misses this portion of the bout distribution. Therefore, r^2^ values remain high, and overall residuals low, despite the sub-optimal single exponential fit. Assessing the normality of the residuals does not offer clarification of this fitting issue, as the distribution of residuals fails normality testing even for a single-exponential fit of 100,000 simulated bout durations drawn from a single exponential distribution (not shown).

Next we compared the single-exponential fits against more complex fits that included the sum of up to 4 exponential functions. For each stage's bout distribution, the fits were compared pair-wise (1-vs-2, 2-vs-3, and 3-vs-4) using a sum-of-squares F-test, as well as Akaike's Information Criteria (AIC) (see methods). For all stage distributions, these two metrics agreed on the best number of exponentials required to fit the distribution. For WASO and NREM distributions, the best fit was obtained with the sum of 3 exponential functions, while for REM, the best fit was the sum of 2 exponentials (Table 2). The fits for NREM sub-stages (NREM1-NREM4) are shown in Table 3, for comparison. Clearly the 3 exponentials of the NREM bouts (sub-stages not considered) is not simply reflective of a sum of mono-exponential substages, since 2-exponential fits were required for NREM1-NREM3. Sample size considerations for fitting of frequency distributions.

**Table 2:** Exponential Fitting Parameters.

	Control	Mild OSA	Severe OSA
**WASO**			
Tau-Fast	0.60 (0.59–0.61)	0.53 (0.53–0.54)	0.53 (0.52–0.53)
% Fast	94.5%	93.2%	97.9%
Tau-Medium	3.1 (2.9–3.2)	2.2 (2.1–2.2)	3.7 (3.6–3.8)
% Medium	5.4%	6.5%	2.1%
Tau-Slow	16.1 (14.1–18.7)	14.6 (13.8–15.5)	n/a
% Slow	0.3%	0.3%	n/a
**NREM**			
Tau-Fast	1.7 (1.6–1.8)	0.9 (0.8–0.9)	1.0 (1.0–1.1)
% Fast	77.3%	75.3%	82.8%
Tau-Medium	7.8 (6.9–9.0)	5.2 (4.9–5.5)	4.8 (4.6–5.1)
% Medium	18.2%	21.6%	15.2%
Tau-Slow	44.1 (40.0–49.1)	37.6 (35.2–40.3)	32.8 (30.6–35.4)
% Slow	4.4%	3.1%	2.0%
**REM**			
Tau-Fast	3.8 (2.8–5.8)	2.6 (2.2–3.0)	1.9 (1.8–2.0)
% Fast	40.5%	57.5%	81.9%
Tau-Slow	19.0 (17.4–20.9)	16.8 (15.8–18.0)	16.3 (15.3–17.6)
% Slow	59.5%	42.5%	18.1%

Tau values (in units of “epochs”) are given as mean with the 95% confidence interval in parentheses.

**Table 3:** NREM sub-stages.

**NREM1**	
Tau-Fast	1.3 (1.1–1.4)
% Fast	93.8%
Tau-Slow	3.6 (2.1–11.5)
% Slow	6.2%
**NREM2**	
Tau-Fast	1.1 (1.1–1.2)
% Fast	90.7%
Tau-Slow	10.8 (10.3–11.4)
% Slow	9.3%
**NREM3**	
Tau-Fast	0.69 (0.67–0.71)
% Fast	94.0%
Tau-Slow	4.3 (4.0–4.6)
% Slow	6.0%
**NREM4**	
Tau	0.79 (0.74–0.84)

Tau values (in units of “epochs”) are given as mean with the 95% confidence interval in parentheses.

**Figure 2:**
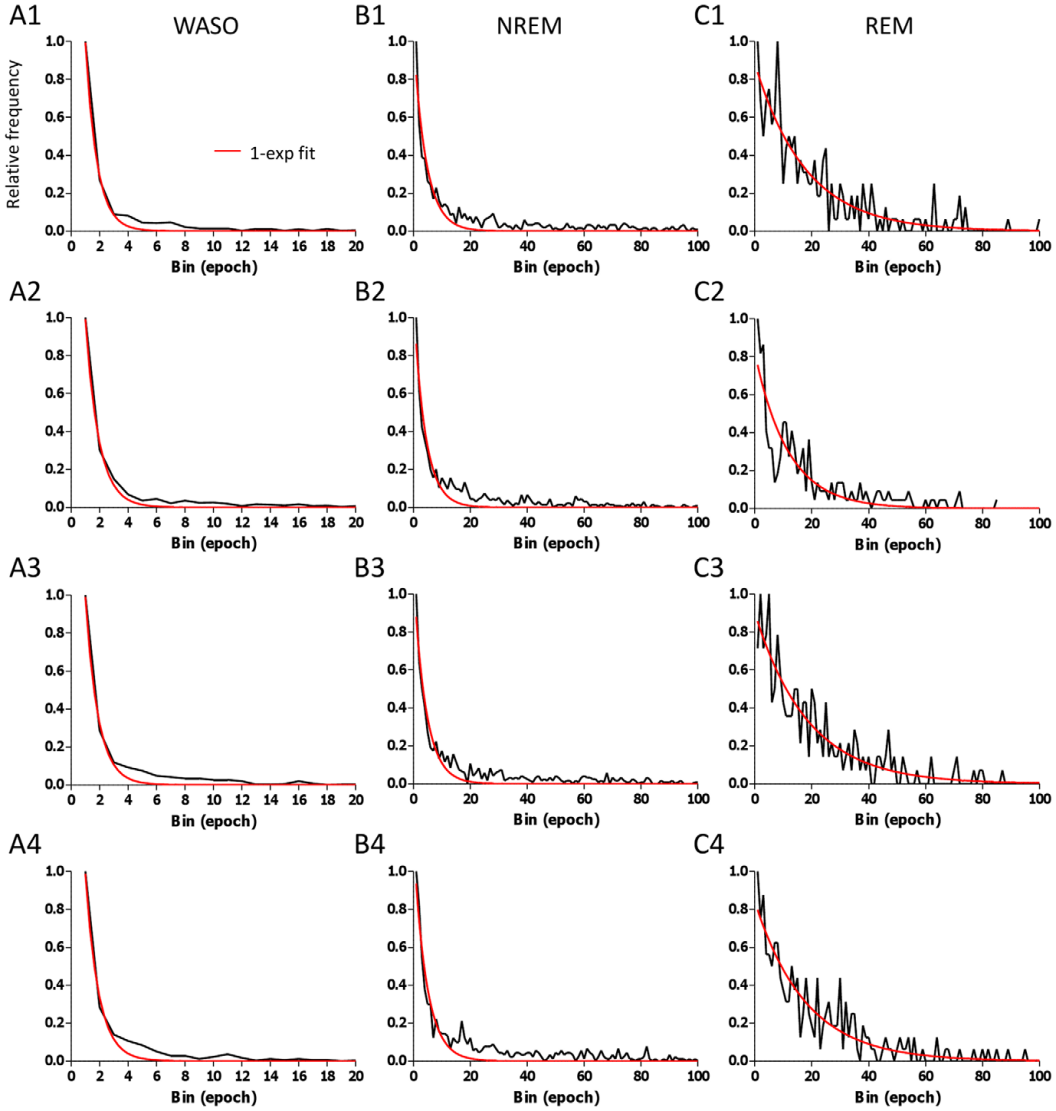
Frequency histograms of random control subgroups. The relative frequency of bouts from four groups of n = 30 randomly chosen individuals selected from the control dataset. Each row represents a different group. The relative frequency of bouts is plotted against the duration of bouts (30-second epoch bins) for WASO (column A), NREM (column B) and REM sleep (column C) bouts. The best single exponential fit is overlaid in red.

We also fit randomly selected subsets of the control group to address the following questions: 1) Does the appearance of multiple exponential functions imply heterogeneity within the control group; and, 2) how different are the resulting fit parameters when the sample size is decreased by more than a factor of 10? The frequency histograms of four randomly selected groups of 30 control individuals are shown in Figure 2. The overlaid best fit single exponential function is visually sub-optimal in these subsets, similar to fits of the full datasets seen in Figure 1. The optimal number of exponentials was 2 (instead of 3 found for the full dataset) for WASO across all four groups. The optimal number of NREM exponentials was 3 for three of the four groups, and 2 exponentials were optimal for the REM distributions across all four groups (similar to the full dataset). The failure to detect the third exponential from the WASO may be related to the very small relative proportion of these long-duration events.

**Figure 3:**
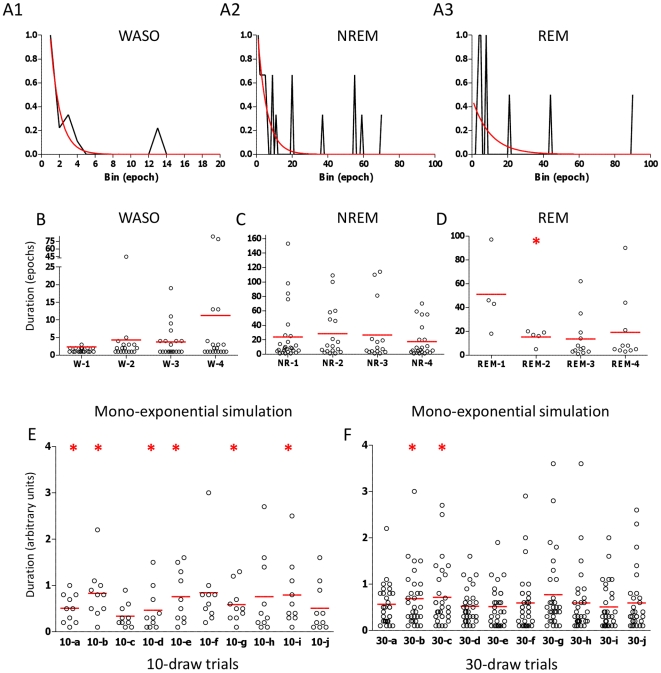
Effects of under-sampling on analysis of bout duration distributions. The relative frequency histograms of WASO (A1) NREM (A2) and REM sleep (A3) bout durations are shown for a single, randomly selected patient from the control group for comparison with histograms from larger samples (Figures 1 and 2). The best single exponential fit is overlaid in red. Bout durations from four randomly selected individuals, are shown in panels B–D, including the single patient shown in panels A1–3 (which corresponds to patient #4 in panels B–D), to illustrate how the distributions can visually or statistically (asterisk) be mistaken as Gaussian. Under-sampling of simulated known monoexponential data leads to common mis-classification of the distribution as Gaussian (E; asterisks), and such mis-classification decreases as the number of samples increases (F).

When only a single night of stage data was considered, the frequency histograms were clearly under-sampled: fitting showed variability between individual patients (Figure 3A), and for REM bouts, convergence was often not possible. The distribution of WASO, NREM, and REM sleep bout lengths is plotted for four randomly selected individuals from the control group, illustrating that variability arises when events are under-sampled (Figure 3B-D). Interestingly, these single-night data sets may even appear to have a Gaussian distribution (Figure 3D). To test this issue of statistical under-sampling of exponential processes, we simulated sleep bout durations drawn from a mono-exponential distribution: 6 of 10 sampling trials passed normality testing when the number of events was 10 per trial (Figure 3E), while only 2 of 10 trials passed normality testing when the number of events per trial was 30 (Figure 3F), and none passed when the number of events per trial was >75 (not shown). These results illustrate a major statistical concern regarding attempts to describe bout distributions with insufficient samples (such as a single night of sleep).

### The effect of OSA on sleep architecture dynamics

**Figure 4:**
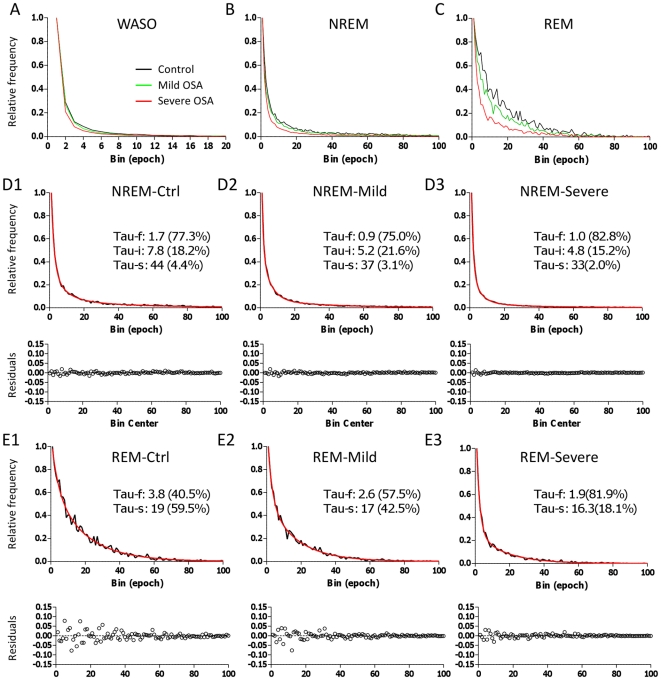
Multi-exponential fits of bout durations and the impact of mild versus severe OSA. Frequency histograms are shown for WASO (A), NREM (B), and REM sleep (C) bouts. Control distributions (black) are compared with those of mild OSA (green) and severe OSA (red). To illustrate visually the goodness of fit, the NREM (row D) and REM (row E) sleep histograms are shown separately, along with the time constants (tau) and % contribution of each exponential function. For NREM sleep, the optimal number of exponentials was three, while for REM sleep, the optimal number was two, regardless of OSA severity. Note the improved residual value patterns, compared to those of the mono-exponential fits from Figure 1.

The optimal number of exponential functions was 3 for NREM, and 2 for REM bout durations, regardless of apnea presence or severity. The optimal exponential fits are shown for NREM and REM bouts across all three cohorts in Figure 4. For NREM and REM bout durations, the analysis detected significant differences not only in the time constants associated with the exponential functions, but also in their relative contributions, as indicated by the y-axis intercept of each component. For example, the faster overall decay of the REM bout distributions with OSA was attributed to faster time constants (particularly of the fast exponential) as well as an increase of the relative contribution of the fast component (at the expense of the slow component). The changes in bout distribution dynamics was not accounted for by a change in the representation of males versus females in the control versus OSA groups (Supplemental Material, Table S1). Although we did not match the groups for other factors such as medications or medical illness, which may also affect sleep architecture, the pattern of REM > NREM fragmentation illustrated by our analysis fits well with the hypothesis that sleep apnea accounts for a large part of the observed changes in transition dynamics.

Given the common practice of fitting single exponential functions to sleep-wake bout duration data, we calculated how the weighted decay time constants (weighted according to their Y_o_ values) from our multi-exponential fits would compare to this technique of fixed single exponential fitting. The weighted time constants of the REM sleep multi-exponential decays were 12.8, 8.6, and 4.5 epochs for control, mild OSA, and severe OSA, respectively (single exponential fits: 14.6, 10.8, 4.8 epochs, respectively). The weighted NREM sleep multi-exponential decays were 4.7, 2.9 and 2.4 epochs, respectively (single exponential fits: 4.2, 3.5, and 2.2 epochs). These weighted decay time constants are similar to the values obtained for the best single exponential fit to the data. OSA clearly affects the best fit mono-exponential time constant (whether by forcing a mono-exponential fit or by calculating a weighted average of a multi-exponential fit). Although mono-exponential fitting can distinguish sleep architecture in the control cohort versus mild OSA and severe OSA, our intention here is not to suggest exponential fitting as a diagnostic tool for detecting OSA, but rather to illustrate the complex dynamics underlying sleep fragmentation, using OSA as a prime example of such pathological architecture.

### Fitting frequency histograms with the power law function

**Figure 5:**
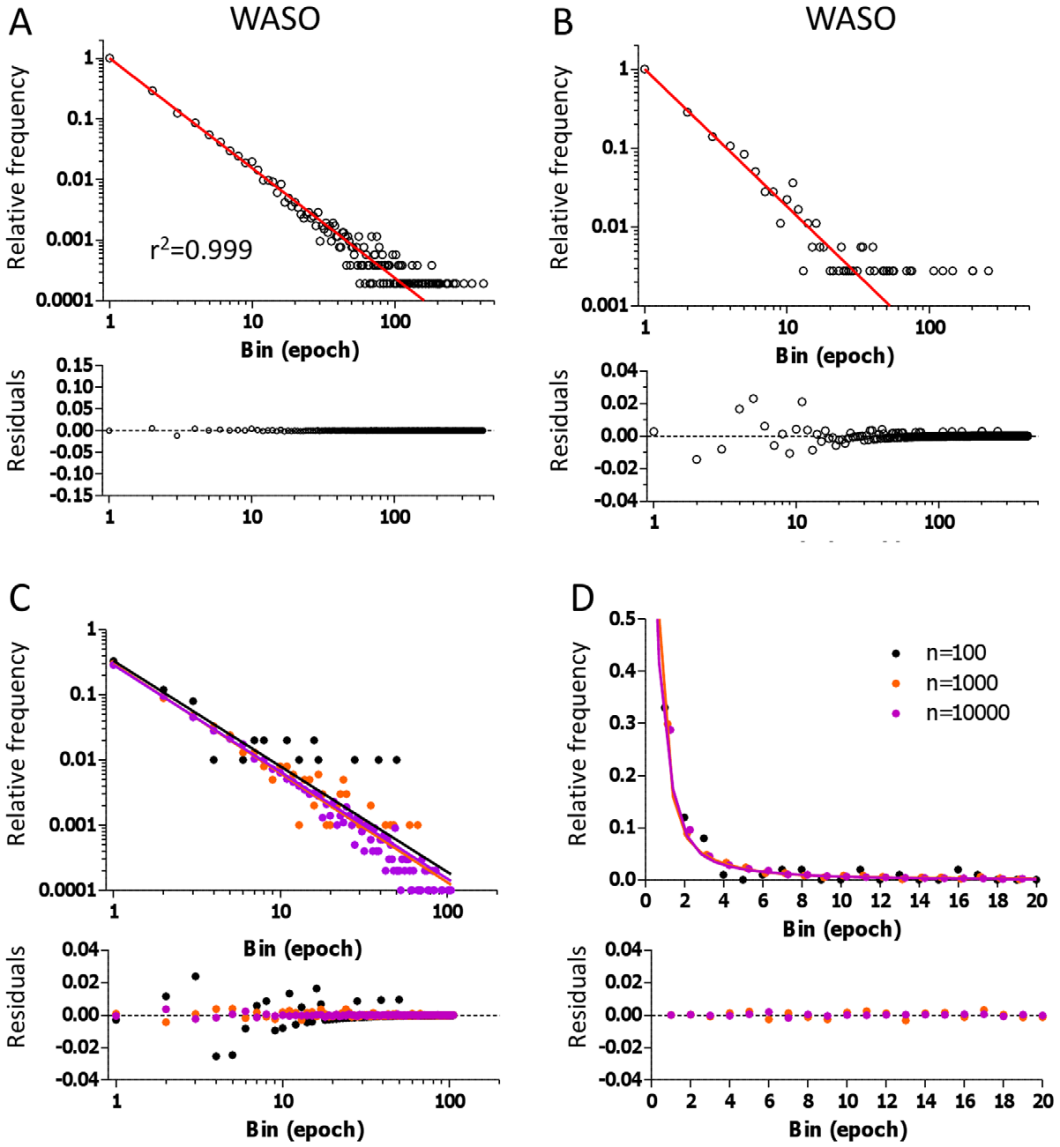
Power Law analysis of WASO bout distributions. The WASO frequency histogram from the control group is shown in log-log display (A), with the fitted power law overlaid in red. A 30-patient subset of WASO is shown in panel B for comparison. Various size samples drawn from three simulated exponential distributions (with time constants of 1, 5, and 25 epochs, chosen to produce relative contributions in exponential fitting of ∼95% fast, 4% intermediate, and ∼1% slow) are shown in log-log plot (C) and linear plots (D) for comparison of exponential and power law fitting.

Several groups have reported that wake bout durations are best described by a power law[19], [20], [22]. We therefore performed power law fitting of the control and OSA frequency histograms of WASO (Figure 5). While a linear appearance on log-log plotted data suggests a power law process, such linearity should be considered necessary but not sufficient. For example, although a single exponential decay appears downwardly convex on a log-log plot (and linear on a semi-log plot), a multi-exponential process may appear linear on a log-log plot. This can be seen in the WASO bout distributions, for the entire control population (Figure 5A) and a randomly chosen 30-patient subset (Figure 5B), which appear linear on a log-log plot but are also well-fit by a 3-exponential process. However, the true underlying distribution of wake bouts is of course not known. Therefore, given the uncertainty, we generated simulated bouts whose lengths were drawn from three known exponential distributions to answer the question: can a known multi-exponential function appear linear (that is, power-law-like) on a log-log plot?

Parameters were chosen to imitate the actual time constants and relative proportions seen in the fitting of the WASO distributions in the control population. This simulation procedure was repeated using three sample sizes that differed by a factor of 10, the largest of which was similar to the total pooled sample size for WASO in the control group. This dataset visually resembled a power law distribution, appearing linear on the log-log plot shown in Figure 5C. The power-law fitted function is also shown in Figure 5C, and the 3-exponential fit is shown for comparison in Figure 5D. Formal comparison between a power law and 3-exponential function revealed that the 3-exponential function was favored for n = 1000 and n = 10,000 samples, while the best function was ambiguous for n = 100. For the comparison between a power law and the sum of two exponential functions, a power law was favored for n = 100 and n = 1000 (two-exponential was favored for n = 10,000). The sum-of-squares and AIC methods differed in some cases, a testament to the potential ambiguity associated with choosing between different fitting functions, even when the samples were known to be drawn from exponential distributions.

## Discussion

This study complements and extends previous work on the sleep-wake dynamics in several respects. First, sleep-wake state transition probabilities are more complex than previously recognized. The temporal stability of NREM and REM sleep clearly requires more than a single-exponential function to describe the bout distributions [19], [20], [23]. Second, our simulations show that multi-exponential distributions may mimic a power law distribution, the typical function used to describe wake bout durations[19], [20], [22]. Third, we demonstrate that one night of data is not an adequate sample of sleep-wake transitions to assess transition dynamics statistically using this distribution fitting method. Finally, we show that sleep fragmentation seen in OSA involves accelerating the rate of “decay” of NREM and REM sleep bout durations.

### Clinical considerations regarding sleep stage transition dynamics

Although it is common for sleep stages to be presented as the average duration of time spent in wake, REM, or NREM sleep stages, metrics such as mean and median may not be informative if the distributions are not Gaussian, particularly if they are highly nonlinear such as exponential or power law distributions. From a “biomarker” standpoint, the pattern and timing of stage transitions may provide clinical insight into fundamental questions about what it means to have “refreshing” sleep than summary stage metrics, although this speculation remains to be tested. REM sleep and SWS have been implicated in different types of learning and memory[24], [25], although the correlations of percentages of these or other sub-stages with subjective daytime symptoms or objective sleepiness is typically modest when present at all, as discussed above. One possibility is that the pattern of transitions is important, and accurate characterization of stage stability (by virtue of bout duration or transition probability fitting) is an important step in this direction. Given the wide spectrum of subjective daytime symptoms (and poor correlation with objective MSLT data) across different degrees of OSA[5], [7], it is worth considering these alternative tools to evaluate PSG data.

Whether different types of fragmentation occur in different pathological states, or with different clinical symptoms, remains to be explored. Although most of the published bout duration analysis has focused on the presence or absence of OSA, recent data suggests that sleep stage stability may be associated with daytime symptoms in populations with syndromes of fatigue or pain[16], [17]. Non-refreshing sleep is a common complaint, with many potential etiologies spanning medical, neurological, and psychiatric domains[26], [27]. Although initially proposed decades ago, there is renewed interest in transition-based approaches to quantify sleep architecture [14], [15]. Indeed, if there are different subtypes of fragmentation, or stage transition patterns are important, summary PSG measures will miss these clues. Here, and elsewhere[14], [15], the pattern of stage stability is clearly different in patients with or without OSA – despite minimal differences apparent in summary statistics such as stage percentages. From a fitting standpoint, our results demonstrate that standard mono-exponential functions do not capture the bout distribution dynamics of WASO, NREM or REM sleep. One interpretation of the multi-exponential process is that there is a balance between sleep stability and sleep satiation. For example, some degree of instability is evident in the proportion of brief events in the fitting, even in healthy control subjects. Longer duration exponentials, in contrast, reflect more stable persistence of state. Satiation results in the culmination of stable sleep state by increasing the tendency to awaken over time.

Our results also emphasize the requirement for sampling far more than one night of sleep to adequately quantify bout duration distributions. Cost and inconvenience prohibits more than one or two nights of sleep in the laboratory setting for individual patients. Whether improvements in home monitoring can offer an alternative, which would allow longitudinal assessments of sleep architecture for individual patients, remains to be explored. Although the within-subject variability is likely less than between-subject variability, the small number of transitions per night suggests the importance of extended monitoring, likely in the home setting.

### Statistical considerations regarding bout duration analysis

Statistical analysis of sleep stage percentages typically assumes a Gaussian distribution, but some studies report mono-exponential distribution of sleep bout durations [19], [20]. We tested the possibility that multiple exponential processes describe sleep-wake stage distributions. The implication of multiple exponential functions describing these distributions is that multiple transition probabilities are involved. Although the “optimal” number of exponentials depends on the sample size and other statistical considerations, clearly the mono-exponential distribution is insufficient. As seen at the biochemical level (for example, in enzyme conformation switching or ion channel gating), the duration of time spent in an observed or “phenotypic” state (such as visually scored REM sleep) may demonstrate multiple underlying rules or “generator” processes (such as may be revealed by multi-exponential fits). Consider by analogy two coins, one that is fair (heads arising with 50% probability), and one that is unfair (say, 90% chance of heads), but the heads and tails of each coin appear visually identical. Because each phenotypic state (heads or tails) has two generators (one for the fair coin, one for the biased coin),the distribution of repeated observations will include contributions of both generator rules. In the same way, a visually identified sleep stage (phenotypic state) may in fact be governed by more than one generator process. Failure to recognize this distinction (such as occurs when one employs a probability matrix[23]) is equivalent to forcing a mono-exponential fit, and may limit the potential ability to map fragmentation patterns to clinical symptoms or pathology. Note that, in the case of NREM bout durations, the 3-exponential fitting did not result from a simple addition of mono-exponential NREM sub-stages; in fact our data suggest that these sub-stages may themselves be governed by multiple generators.

Our results identify an important statistical limitation in the commonly employed r^2^ value, which reports excellent (>0.9) values despite largely missing the long tails of the distributions. Moreover, analysis of residuals between the fitted curve and the actual data, often used to test the goodness of fit, also under-weights the poor fitting evident in the long tail events. This occurs because of the relatively low probability of long events, yielding small residual values even for forced mono-exponentials that miss long events (see Figure 1).

Finally, although there is ongoing interest in fitting wake bouts to a power law distribution, the distinction between a power law and a multi-exponential distribution is not always straightforward. Indeed, simulations showed that a known multi-exponential process can visually and statistically resemble a power law. This has mathematical implications for sleep transition modeling. For example, if all sleep-wake bout durations are considered to be exponential or multi-exponential, then all transitions of the hypnogram may be simulated using a Markov chain model. Although there are several limitations, the appeal of Markov models is that stage transitions are considered probabilistic, and certain transitions may be stabilized or destabilized by different neural circuits or neuromodulators [22], [28], [29].

### Physiological implications for exponential bout durations

The transition between sleep and wake (and between REM and NREM sleep) has been compared to a “switch” consisting of reciprocal inhibition between neurons whose firing favors one or another state[2], [30]. Indeed, optogenetic stimulation of orexin neurons in transgenic mice increased the probability of transition from sleep to wake[31]. Whether detailed transition analysis of existing animal lesion studies targeting specific sleep- or wake-promoting nuclei can shed additional light on the neural circuitry underlying the transition probability dynamics remains to be seen.

From the standpoint of future sleep architecture “fingerprinting”, there is potential for use of Markov chain models[23], [32], [33], the parameters of which could be extracted from sleep architecture information. For example, disease states (or lesion sites) could be associated with changes in the number of exponential functions describing a given stage distribution, which stage transitions are possible, and the probabilities governing each transition. Regarding the homeostatic and circadian influences on state transition probabilities, analysis of sleep dynamics in humans subjected to forced desynchrony protocols may prove important. The ultimate goal is to link sleep architecture patterns to anatomical, physiological, behavioral, and pathophyisological aspects of sleep and wake function.

### Conclusion

Clinical correlations between daytime complaints and polysomnographic metrics of disease severity are not always straightforward, due in part to inter-subject variability, largely subjective complaints, variable intrinsic tolerance to sleep disruption, and the short duration and non-natural setting of routine clinical monitoring. Even OSA-mediated fragmentation can be missed in routine clinical metrics (such as stage percentages). The key concept is that seemingly complex and variable manifestations of “fragmentation” may in fact possess objective and identifiable underlying statistical structure, which may offer opportunity for improved correlation with clinically relevant endpoints.

## Materials and Methods

### Database cohorts

Three groups of patients were selected from the Sleep Heart Health Study, a large database of home-based polysomnography (PSG) in over 6000 patients[34]. We have obtained Category IV Institutional Review Board (BIDMC) approval for use of the data obtained from this database, the data of which is anonymous and thus we do not require additional consent. The pre-defined groups included controls (defined as AHI<5, ESS<10, no medications and no cardiovascular co-morbidities), and two groups with sleep disordered breathing: mild OSA (AHI 5–15), and severe OSA (AHI >30). The duration of time spent in any stage, measured in units of 30-second epochs, was analyzed for each group, including wake (after sleep onset; WASO), NREM1, NREM2, NREM3, NREM4, and REM sleep stages. We also considered NREM as a single stage in separate analyses (ignoring transitions between NREM sub-stages). Note that for the clinical characteristics in Table 1, we used the accepted clinical definitions of NREM sleep sub-stages (N1-N3), but for the exponential fitting, we used the traditional 4 stage classification, as originally reported in the SHHS. Note also that we did not control statistically or attempt to match the cohorts initially for differences in age, sex, medical comorbidities, or medications (as was done for example in Swihart et al[15]). Differences in these factors are illustrated in Table 1 (using Chi^2^ or ANOVA as appropriate). Because of the larger difference in M:F proportion across groups, we did re-analyze the exponential fitting of the control cohort by sex (Supplemental Material, Table S1). Although small differences are evident, the change in proportion of M:F in the OSA groups does not account for the observed differences reported in the main text. In particular, for example, the REM bout durations, which show the most marked impact of OSA, had similar parameters.

### Bout duration analysis and curve fitting

All bouts of a given stage from each subject groups were combined for statistical analysis. Frequency histograms of bout durations were generated by Prism (GraphPad Software, LaJolla, CA, USA). Bin width was one epoch. The relative frequency of bouts in each bin was calculated, and the resulting histograms were normalized to the maximal relative frequency (which was always in the shortest bin) before fitting routines were undertaken. All stage distributions, in each clinical group, failed three tests of normality (D'Agostino-Pearson, Shapiro-Wilk, and Kolmogorov-Smirnoff). Each curve was fitted first with a standard exponential decay function: Y  =  Y_o_ * e^−kX^ + C, where *Y_o_* is the Y-intercept value, *k* is the rate constant describing the rate of decay of the function, *X* is the time (in units of epochs), and *C* is a plateau value (which we forced to zero). Fitting constraints included positive *k* values (required for a decay), and zero C value because there is no biological reason to consider otherwise. For multiple exponential fits, the equation involves a linear sum of *i* components defined by [Y_o*i*_ * e^−k*i*X^] values, the *Y_o_* of which corresponds to the intercept of each component. Although we refer to this term as the “relative contribution” of that component, it reflects neither the number of events associated with that time constant (which can far exceed the coefficient proportion), or the area under the curve of that exponential decay (which is biased against fast time constants). Confidence intervals are shown to allow comparison of each parameter between the three cohorts.

Goodness of fit was compared between the best single-exponential function and the sum of *i*  = 2, 3 or 4 exponentials, using in each case a non-linear sum-of-squares F-test (which considers how well a fitted curve matches the data) and Akaike's Information Criteria (AIC) (which considers which of two functions fits the data better, but does not consider the goodness of fit *per se*), using built-in Prism routines. No weighting of residuals was implemented (exponential fits did not converge if Y-value weighting was used). Each function's goodness of fit was compared sequentially: 1 versus 2 exponential components, then 2 versus 3, and so forth, until the additional component no longer significantly improved the fit by F-test criteria, or the algorithm failed to converge within 3000 iterations. Higher numbers of exponentials were not tested because the sum of 4 exponentials never provided a significantly better fit than 3 exponentials. The optimal exponential fit was then compared with the fit provided by a power law: Y  =  A * X^B^, where *A* is a constant, *X* is time (in units of epochs), and *B* is the “critical” or scaling exponent characterizing the power law. This was also subject to both non-linear sum-of-squares, and AIC criteria. Note that Prism fits the function to the actual data, rather than fitting a linear regression to the semi-log or to the log-log plotted data.

Simulations: The only simulated data appears in Figures 3 (D, E) and 5. To generate simulated bout lengths, we used MatLab (MathWorks, Natick, MA, USA): the “exprnd” function is a random number generator following a single exponential distribution specified by a time constant of decay; the “randsample” function was used to draw from the generated distributions. These simulation bout lengths were exported for analysis in Prism as above.

## Supporting Information

Table S1 (0.04 MB DOC)
