# Supplementary material for: Obstructive Sleep Apnea Alters Sleep Stage Transition Dynamics
Source: PLoS One. 2010 Jun 28;5(6):e11356. doi: 10.1371/journal.pone.0011356 (PMC2893208; doi:10.1371/journal.pone.0011356)
Supplement: Table S1 — (0.04 MB DOC) [file pone.0011356.s001.doc]

**Table S1. Exponential Fitting Parameters: Male vs Female, control group**

|  | Male | Female |
| --- | --- | --- |
| **WASO** |  |  |
| Tau-Fast | 0.63 (0.62-0.65) | 0.57 (0.56-0.58) |
| % Fast | 94.1% (93.6-94.7) | 94.2% (93.8-94.7) |
| Tau-Medium | 3.2 (2.9-3.5) | 2.6 (2.4-2.8) |
| % Medium | 5.7% | 5.2% |
| Tau-Slow | 24.4 (14.9-33.9) | 10.7 (9.2-12.1) |
| % Slow | 0.18% (0.09-0.26) | 0.58% (0.43-0.72) |
| **REM** |  |  |
| Tau-Fast | 6.5 (3.3-211.3) | 2.6 (2.0-3.6) |
| % Fast | 42.1% (0-99.2) | 45.9% (41.3-50.6) |
| Tau-Slow | 16.5 (11.7-28.5) | 21.1 (19.7-22.7) |
| % Slow | 57.9% | 54.1% |
| **NREM1** |  |  |
| Tau-Fast | 1.4 (1.3-1.5) | 1.2 (1.1-1.4) |
| % Fast | 94.8% (85.5-100) | 91.6% (82.0-100) |
| Tau-Slow | 4.1 (2.3-19.8) | 3.3 (2.0-11.5) |
| % Slow | 5.2% | 8.4% |
| **NREM2** |  |  |
| Tau-Fast | 1.2 (1.1-1.2) | 1.1 (1.1-1.2) |
| % Fast | 88.6% (87.9-89.3) | 91.6% (91.2-92.0) |
| Tau-Slow | 10.8 (10.2-11.6) | 10.9 (10.4-11.4) |
| % Slow | 11.4% | 8.4% |
| **NREM3** |  |  |
| Tau-Fast | 0.65 (0.62-0.68) | 0.71 (0.69-0.73) |
| % Fast | 94.7% (94.1-95.3) | 93.8% (93.3-94.3) |
| Tau-Slow | 4.05 (3.67-4.51) | 4.4 (4.1-4.7) |
| % Slow | 5.3% | 6.2% |
| **NREM4** |  |  |
| Tau | 0.63 (0.59-0.67) | 0.83 (0.78-0.89) |

Tau values (in units of “epochs”) are given as mean with the 95% confidence interval in parentheses.
